# Bioconversion of Cheese Whey and Food By-Products by *Phaeodactylum tricornutum* into Fucoxanthin and n-3 Lc-PUFA through a Biorefinery Approach

**DOI:** 10.3390/md21030190

**Published:** 2023-03-19

**Authors:** Giovanni Luca Russo, Antonio Luca Langellotti, Vito Verardo, Beatriz Martín-García, Maria Oliviero, Marco Baselice, Prospero Di Pierro, Angela Sorrentino, Sharon Viscardi, Luis Marileo, Raffaele Sacchi, Paolo Masi

**Affiliations:** 1 CAISIAL Center, University of Naples Federico II, Via Università 133, 80055 Portici, Italy; langello@unina.it (A.L.L.);; 2 Department of Nutrition and Food Science, Campus of Cartuja, University of Granada, 18071 Granada, Spain; 3 Institute of Nutrition and Food Technology ‘José Mataix’, Biomedical Research Center, University of Granada, Avda del Conocimiento sn., 18100 Granada, Spain; 4 Department of Animal Health, Experimental Zooprophylactic Institute of Southern Italy, Via Salute, 2, 80055 Portici, Italy; 5 Department of Civil, Environmental, Land, Construction and Chemistry (DICATECh), Politecnico di Bari, 70126 Bari, Italy; 6 Department of Agricultural Sciences, Unit of Food Science and Technology, University of Naples Federico II, 80055 Portici, Italy; 7 Biotechnology of Functional Foods Laboratory, Camino Sanquilco, Parcela 18, Padre Las Casas 4850827, La Araucanía, Chile; 8 Núcleo de Investigación en Producción Alimentaria, Universidad Católica de Temuco, Rudecindo Ortega 02950, Temuco 4780694, La Araucanía, Chile; 9 Programa de Doctorado en Ciencias Agropecuarias, Facultad de Recursos Naturales, Universidad Católica de Temuco, Rudecindo Ortega 02950, Temuco 4813302, La Araucanía, Chile

**Keywords:** PUFA, sustainability, food by-product, bioconversion, biorefinery, dairy wastewater, microalgae

## Abstract

This study investigates the potential of utilizing three food wastes: cheese whey (CW), beet molasses (BM), and corn steep liquor (CSL) as alternative nutrient sources for the cultivation of the diatom *Phaeodactylum tricornutum*, a promising source of polyunsaturated eicosapentaenoic acid (EPA) and the carotenoid fucoxanthin. The CW media tested did not significantly impact the growth rate of *P. tricornutum*; however, CW hydrolysate significantly enhances cell growth. BM in cultivation medium enhances biomass production and fucoxanthin yield. The optimization of the new food waste medium was conducted through the application of a response surface methodology (RSM) using hydrolyzed CW, BM, and CSL as factors. The results showed a significant positive impact of these factors (*p* < 0.005), with an optimized biomass yield of 2.35 g L^−1^ and a fucoxanthin yield of 3.64 mg L^−1^ using a medium composed of 33 mL L^−1^ of CW, 2.3 g L^−1^ of BM, and 2.24 g L^−1^ of CSL. The experimental results reported in this study showed that some food by-products from a biorefinery perspective could be utilized for the efficient production of fucoxanthin and other high-added-value products such as eicosapentaenoic acid (EPA).

## 1. Introduction

The cultivation of microalgae is widely diffused for the production of valuable chemical compounds, including natural pigments, fatty acids, biofuels, and dietary supplements [1]. Despite the many advances obtained in recent years regarding the scalability of cultivation methods, the reduction of production costs, and the increase in productivity of molecules of interest, there are two challenges that require research efforts.

The diatom *Phaeodactylum tricornutum* is a microalga that has been extensively studied for its ability to produce polyunsaturated fatty acids (PUFAs), especially eicosapentaenoic acid (EPA, C20:5(*n*−3)) but also for the production of pigments and antioxidants [2,3]. The omega-3 long-chain fatty acids (LC-PUFAs) represent an important class of nutritional compounds, largely recognized to be fundamental elements in the human diet, with a series of health effects and for the treatment of cardiovascular diseases, schizophrenia, cancer, and Alzheimer’s syndrome [4,5].

*Phaeodactylum tricornutum* has gained much attention in the last decades due to its ability to grow in large-scale facilities while producing bioactive compounds such as carotenoids with proven benefits for human health [6]. A wide range of carotenoid pigments, in particular fucoxanthin and β-carotene, have been reported to be efficiently produced by *P. tricornutum* [7]. Fucoxanthin is a xanthophyll produced by brown macro and microalgae. The production of fucoxanthin from microalgae is considered to be the most cost-effective method due to their higher intracellular contents (2–60 mg g^−1^ of DW) [8]. In fact, *P. tricornutum* biomass contains at least ten times more fucoxanthin per gram of dry weight than brown algae [9]. However, the optimization of yields needs to be further achieved.

*P. tricornutum* showed good metabolic versatility, being able to use different types of nutrients and grow with food waste-based media. In fact, it is capable of growing under mixotrophic conditions using a variety of organic compounds [10].

In recent years, this microalga has been cultivated using kitchen wastewater [11], food waste digestates [12,13], food waste hydrolysate [14], citric acid and effluents from sugar and tannery industries [15], and crude glycerol [16,17]. Food by-products and waste (FBW) are side-streams characterized by high amounts of organic carbon, proteins, and mineral salts, which could be usefully recovered for biomass cultivation [2,18]. Actually, some FBW have been successfully used for the cultivation of microorganisms and the cheapest ones are sugar molasses, CSL, whey permeate (WP), and glycerol. Dairy industry by-products and waste, such as cheese whey and other effluents, have been extensively studied in recent years as a new nutrient source for the cultivation of many algae and protists [19,20,21]. However, the costs related to the pre-treatments of food waste can affect the economic advantage of using this waste instead of bulk materials [22]. For this reason, the cultivation of biomass with a high added value is necessary in order to meet production costs and to increase the economic feasibility of the whole process.

To the best of our knowledge, literature data concerning the growth kinetics of *P. tricornutum* using food waste medium are scarce, and, as far as we know, the cultivation with cheese whey or other dairy wastewaters as a nutrient source has never been studied before.

Therefore, the aim of this work was to optimize the growth conditions for *P. tricornutum* using alternative food-waste-based media in order to understand the potential to use cheese whey, molasses, and CSL as nutrient sources for the cultivation of this biomass. The optimization of fucoxanthin production of *P. tricornutum* was studied and reported, comparing the standard medium with the new medium from food industry by-products.

## 2. Results

### 2.1. Characterization of FBW Used and Screening Results

The results of the physicochemical analyses of the FBW used in this paper are reported in Table 1.

The concentrated CW under analysis was rich in protein and lactose (28.8 g/kg and 196.8 g/kg, respectively). The phosphorus content was also very interesting, which was found to be the highest among the food waste analyzed (145 mg/kg). CSL was found to be the richest FBW in terms of proteins, with a concentration of 305 g/kg, but also in lactic acid (121.2 g/kg). Furthermore, it is the FBW that reported very high ash content. BM, on the other hand, is characterized by very high sugar (667 g/kg) and ash content (81.9 g/kg).

**Figure 1:**
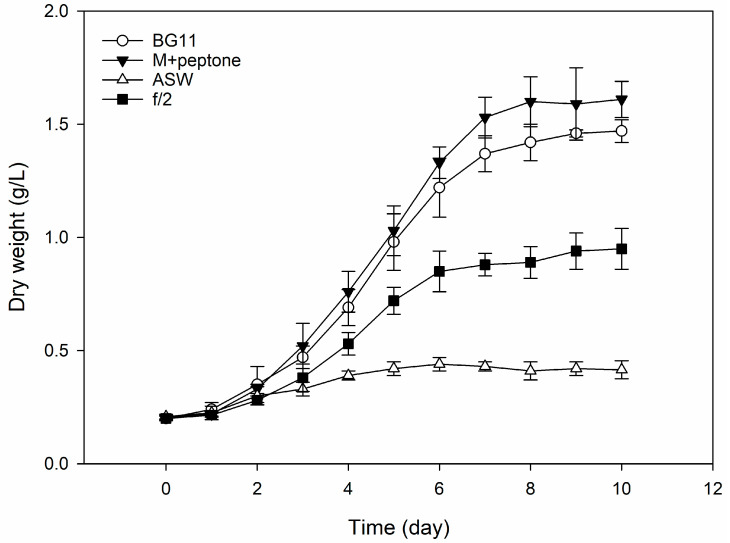
Growth performances of *P. tricornutum* with different cultivation media for 10 days. ASW stands for artificial seawater without nutrients, while M+ peptone refers to BG11 medium with peptone in substitution of NaNO_3_. Values expressed as *mean (n = 3*) ± SD.

Based on the chemical characterization of the FBWs, a screening was performed to assess the growth of *P. tricornutum* on various standard media. Figure 1 reports the growth performances of *P. tricornutum* grown on BG11 medium, Guillard F/2, artificial seawater (ASW, no nutrient added), and BG11 medium with peptone in substitution of NaNO_3_.

Significant differences were observed between the Guillard F/2 medium and BG11. In fact, the latter reported significantly higher productivity with respect to the F/2 medium, reporting a biomass DW after 10 d of 1.5 g L^−1^ in comparison to the 0.87 g L^−1^ of the F/2 medium. The ASW reported no significant growth, only doubling the initial biomass concentration (0.41 g L^−1^).

**Figure 2:**
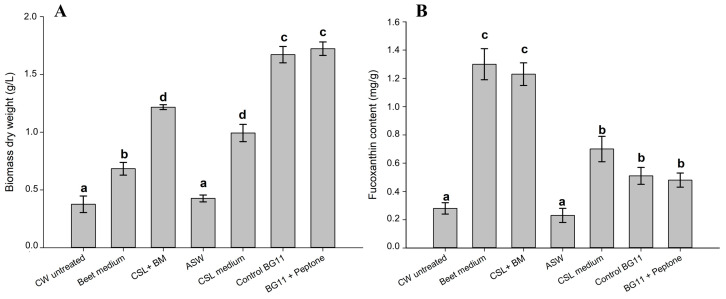
Growth results of *P. tricornutum* using various FBWs (**A**) and amount of fucoxanthin produced after 8 days of cultivation (**B**). Values are expressed as means ± SD (*n* = 3). Different letters mean a significant difference (*p* < 0.05).

The results of FBW medium screening are reported in Figure 2.

The combination of FBW that reported the highest growth was the one with the combination of BM and CSL (Figure 2A). The CW-only-based media did not report significant growth. In fact, they showed a growth similar to that of ASW, and therefore without the addition of nutrients. Moreover, the BM medium also registered low biomass production (0.67 g L) compared to the control.

During the screening, the concentration of fucoxanthin produced from *P. tricornutum* growth with the tested media was also evaluated and is reported in Figure 2B.

In this case, the highest fucoxanthin concentration was detected in the biomass grown with BM and CSL. In fact, significantly higher fucoxanthin content was observed with these FBW-based media compared with standard media. The fucoxanthin content detected in the CW-based medium, on the other hand, is the one that recorded the lowest content.

**Figure 3:**
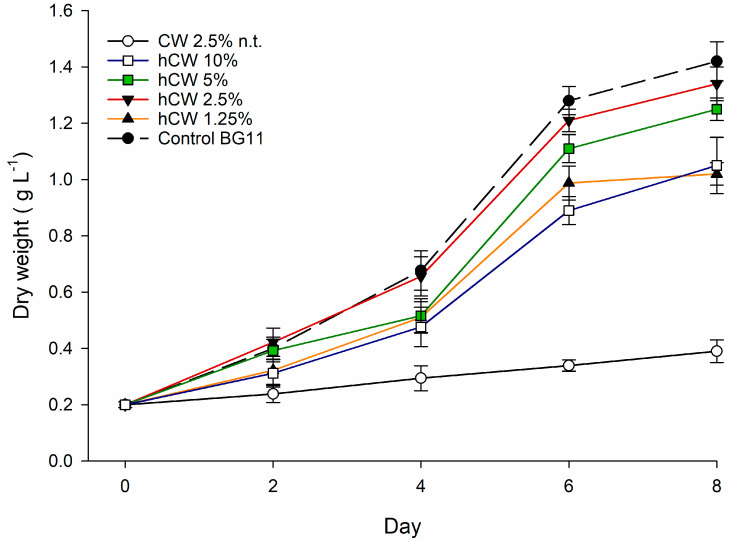
Evaluation of growth performance with untreated CW medium (CW n.t.) and with sequential lactose–protein hydrolysis at different CW concentrations *v*/*v* (hCW 1.25%, hCW 2.5%, hCW 5%, and hCW 10%) in comparison with BG11 media. No nutrients were supplemented to all CW media.

To evaluate the use of CW as a nutrient source for *P. tricornutum*, enzymatic treatment was performed to increase nutrient bioavailability. The growth results using hydrolyzed CW are shown in Figure 3.

After the enzymatic hydrolysis treatment, significant growth of *P. tricornutum* was observed. In fact, with the new hydrolyzed CW-based medium, the growth performances were comparable to those of the control. The optimal concentration of hydrolyzed CW resulted to be the one at 2.5% *v*/*v*, which recorded growths not significantly different from those of the control. At 5% *v*/*v*, slower biomass productivity was observed with respect to the 2.5% *v*/*v* concentration after 4 days of cultivation, but no significant differences were observed in final concentrations. For that reason, in the subsequent RSM–CCD experiment, hydrolyzed CW was used.

### 2.2. Results of Medium Optimization Using RSM–CCD

The results of the RSM–CCD experiments are reported in Table 2.

Table 2 shows the CCD design and the results with the responses. Biomass concentration (expressed as g L^−1^ of DW) and fucoxanthin yield (mg L^−1^) were used as responses and were calculated at the culture log phase (8 days).

The significance of the model for biomass production, derived from the multiple regression analysis of the data, was tested by analysis of variance (ANOVA) and reported in Table 3.

The significance of the model for fucoxanthin yield was also tested by (ANOVA) and is reported in Table 4.

The model fit is also expressed with the coefficient of determination (R^2^) which was 0.9622 for fucoxanthin yield and 0.9712 for biomass dry weight, indicating that more than 95% of the variability in the Y (responses) could be explained by the model. The *p*-value of the models was (*p* < 0.05) which implied that all the models were significant, and also that the lack of fit is non-significant (*p* > 0.05), proving the validity of the model. The experimental results obtained from CCD were regressed using a quadratic polynomial equation, and the regression equations for biomass dry weight (1) and fucoxanthin yield (2) are shown below.

(1) $$\begin{array}{l} Biomass\;DW\left(gL^{-1}\right)=0.016+0.619\;Molasses+0.0561\;CW\\ +\;0.979\;CSL-0.1060\;Molasses\;\times \;Molasses-0.000441\;CW\times CW\\ -\;0.157\;CSL\times CSL-0.00395\;Molasses\times CW\\ -\;0.0534\;Molassesx\;CSL-0.01108\;CW\times CSL \end{array}$$

(2) $$\begin{array}{l} Fucoxanthin\;yield\left(gL^{-1}\right)=-0.175+0.190\;Molasses+0.0528\;CW\\ +\;2.026\;CSL+0.0661\;Molasses\times Molasses-0.000167\;CW\times CW\\ -\;0.148\;CSL\times CSL+0.00227\;Molasses\times CW-0.191\;Molasses\times \\ CSL-0.0322\;CW\times CSL \end{array}$$

The stationary point for this model was reached at 2.24 g L^−1^ of BM, 33.6 mL L^−1^ of CW, and 1.85 g L^−1^ of CSL, which are the best conditions to maximize biomass growth (2.22 g L^−1^) and fucoxanthin yield (3.44 mg L^−1^) for *P. tricornutum*.

**Figure 4:**
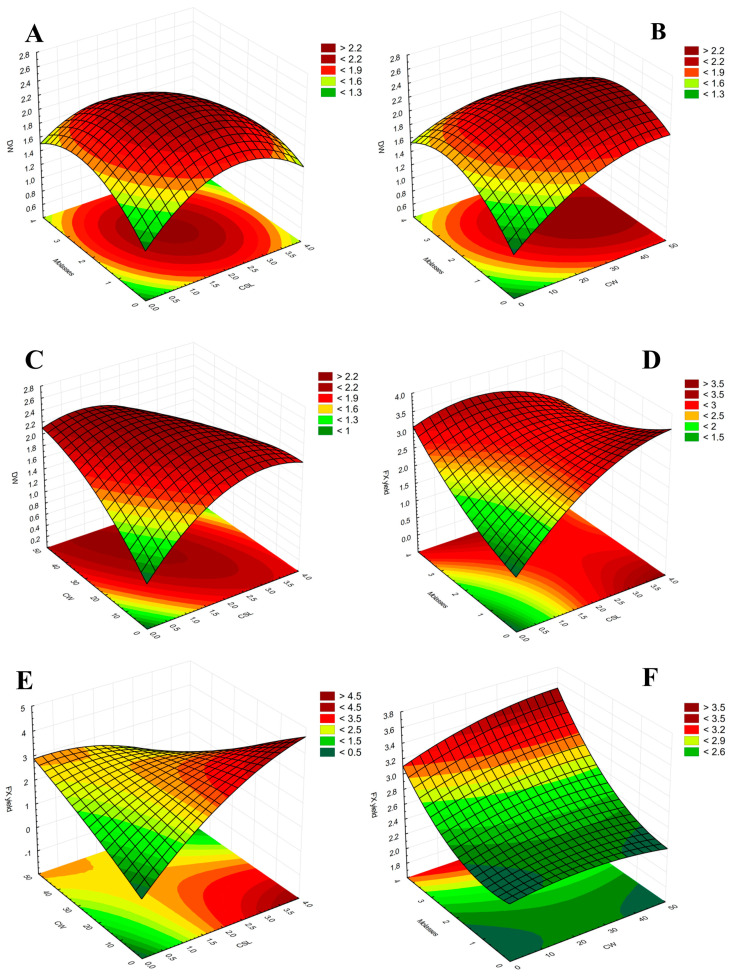
Surface and contour plot (**A**–**F**) of CCD experiments showing the combined effects of the three factors used, beet molasses, cheese whey, and corn steep liquor (CSL), as a function of relative response: biomass dry weight (DW, g L^−1^) and fucoxanthin (FX) yield (g L^−1^).

In order to visually evaluate the results obtained from the RSM–CCD, 3D surface and contour plots were elaborated and reported in Figure 4.

The surface-contour plot reported represents the variation in the experimental responses attained with multiple combinations of two independent variables while holding one of the components constant in the second-order polynomial model.

### 2.3. Fatty Acids Analysis and Carotenoid Profile

The carotenoid profile of *P. tricornutum* grown with different media is reported in Table 5.

The carotenoid profile of the new optimized medium was compared with the control and another FBW-based medium (without optimization). The highest fucoxanthin content was achieved with the medium optimized after RSM–CCD. The optimized medium obtained a fucoxanthin yield of 3.64 mg L^−1^ and the highest β-carotene content (47.6 µg/g). The obtained yields were in line with the predicted values of the RSM model.

**Figure 5:**
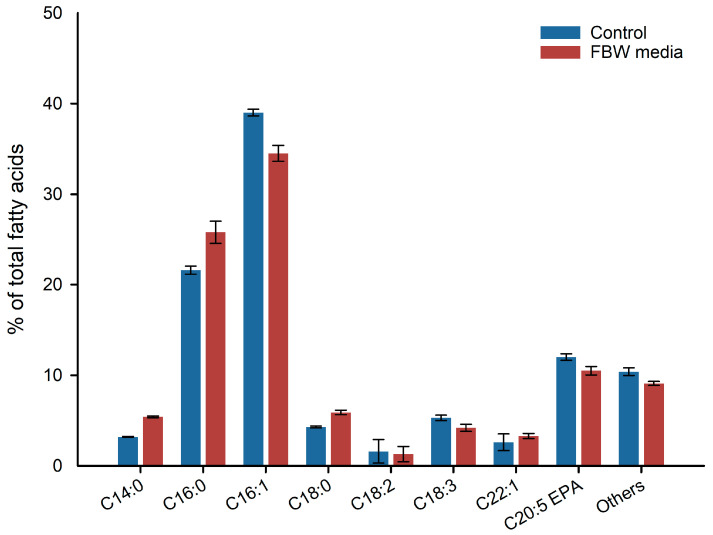
Fatty acid profile of extracted lipid from *P. tricornutum* cultivated using new food by-products optimized media and standard media (BG11). The extraction was performed on biomass after 8 days of cultivation. Values are expressed as means ± SD (*n* = 3).

On the other hand, Figure 5 shows the fatty acid profile of *P. tricornutum* grown with BG 11 (control) and optimized food-waste-based medium.

No significant differences were detected between the control and the new optimized medium after RSM–CCD. Both cultures recorded an EPA content between 11 and 13% of total fatty acids (TFA). The predominant fatty acid was palmitoleic acid, with a concentration between 34 and 38% on TFA, followed by palmitic acid.

## 3. Discussion

In this paper, the effect of an FBW-based medium on the biomass and fucoxanthin productivity of *P. tricornutum* was studied considering different concentrations of CW, CSL, and BM.

The chemical-physical characterization of FBW carried out in this study (Table 1) revealed a high sugar content for CSL and BM, while the CW analyzed reported a good content of proteins and organic nitrogen. The CSL and BM characterization is in line with the literature data [23,24].

In our case, we tested various standard growth media first, in order to select the optimal standard media for *P. tricornutum* cultivation trials. Guillard F/2 medium is largely used as a reference medium in many studies on *P. tricornutum* [13], while BG11 medium is usually used to boost the microalgal biomass production [25]. Moreover, we evaluated the substitution of sodium nitrate in BG11 medium with a source of organic nitrogen (peptone) in order to understand the effect of organic nitrogen on *P. tricornutum* growth. In fact, organic nitrogen is usually found in FBW such as CW and CSL in form of proteins, small peptides, and amino acids [19].

As shown in Figure 1, the BG11 medium showed a significant impact on biomass productivity, doubling the biomass productivity with respect to the Guillard F/2 medium. This could be attributed mainly to the difference in nitrogen content between the two media (0.2 g L^−1^ for BG11 and 0.075 g L^−1^ for F/2 medium). Therefore, we used the BG11 medium as a reference for further experiments.

Furthermore, the BG11 medium with organic nitrogen achieved the highest biomass productivity, even if not significantly different from standard BG11. This is in agreement with the study by Yuan et al., 2020 which reported a significant productivity increase in *P. tricornutum* with organic nitrogen supplementation (spent yeast) [26]. Wheeler et al. (1974) reported that *P. tricornutum* could utilize amino acids as organic substrates (Ala, Glu, Gly, Ser, and Orn) to sustain its growth [27]. This is a positive finding as CW and molasses are usually rich in residual proteins and amino acids [2].

*Phaeodactylum tricornutum* is a diatom that has shown a high capacity for use as a biological platform for the production of high-added-value products [2,3]. In particular, it is reported in the scientific literature that this microalga is able to grow not only in autotrophy but also in conditions of mixotrophy [28,29]. Compared to the autotrophic culture, the mixotrophic culture enhances the cell growth rate and biomass production of *P. tricornutum* [30]. However, it is not clear whether this affects the productivity of the pigments in the biomass. In fact, in the work of Liu et al. (2009), a lower pigment content is reported for mixotrophic cultures compared to autotrophic ones [30]. By contrast, the work of Yang et al. (2020) reports a significantly higher content of fucoxanthin in mixotrophic cultures of *P. tricornutum* compared to those in autotrophy [28].

Once the ability to use organic substrates was established, we tested various FBWs as alternative growth media. The media formulated from BM and CSL showed significant growth, but lower if compared to the control. However, the CW-based medium did not show any growth. For this reason, we attempted to hydrolyze the CW to increase the bioavailability of the nutrients. In fact, after the hydrolysis, significant growth of *P. tricornutum* was observed, which was also comparable to the control (Figure 3). This is in line with our previous study where a significant growth boost for an aquatic protist was observed after the hydrolysis of a dairy wastewater [19]. We observed that the highest biomass concentration was obtained at 2.5% concentration of hydrolyzed CW. However, after 8 days of cultivation, the biomass concentration was not significantly different from the 5% *v*/*v* of CW. For that reason, we evaluated the optimal concentration of CW in the subsequent RSM–CCD experiment. Regarding fucoxanthin production, we observed that this was significantly higher when *P. tricornutum* was grown on BM- and CSL-based media. In fact, with the CW-based media, the fucoxanthin content was lower than the control. In scientific literature it is reported that a decreased light penetration could help to boost fucoxanthin and EPA production in *P. tricornutum* [26]. It is reported that the genes encoding enzymes in the biosynthesis of geranylgeranyl diphosphate (GGPP), which is the precursor of both Chl *a* and fucoxanthin, were downregulated upon high light intensity in *P. tricornutum* [31]. In our case, the media with BM and CSL appears with a brownish color, which can effectively hinder the penetration of light into the culture. This could explain the higher fucoxanthin content with respect to CW which instead gave a light coloring to the growth medium. Furthermore, it is reported that a saline increase in marine microalgae cultivation medium could affect carotenoid production [32]. In fact, in a recent study, the optimal saline concentration of *P. tricornutum* was evaluated. The authors concluded that a concentration of 45 g L^−1^ positively affects the fucoxanthin content and biomass productivity with respect to the standard 35 g L^−1^ for seawater [32]. Therefore, BM and CSL, which are rich in mineral salts (Table 1), increase the saline content of the medium, thus promoting the productivity of fucoxanthin.

In order to evaluate the optimal fucoxanthin productivity using the selected FBW, we performed an RSM–CCD using CW, CSL, and BM as factors.

The combination of the three FBW has proved to be a good choice to have both high biomass productivity and a good fucoxanthin yield. In fact, a high content of fucoxanthin can often affect the productivity of the biomass [28]. In our case, the results obtained after the CCD confirmed the screening tests in terms of fucoxanthin productivity, demonstrating that adding BM and CSL is significant for increasing the fucoxanthin concentration of *P. tricornutum.* CW, on the other hand, is an important element for the new media in order to sustain biomass production, but it is not significant for the improvement of fucoxanthin content. This trend is more understandable through the surface-contour plots reported in Figure 4.

In the work of Yuan et al. (2020) [26] the spent brewery yeast was used as an alternative nutrient ingredient for a sustainable medium for *P. tricornutum*. The authors also reported the feasibility of using an FBW for the obtainment of fucoxanthin. Compared to our results, we obtained a higher biomass concentration (2.2 g L^−1^) with respect to the latter work (less than 1 g L^−1^), but a lower fucoxanthin yield (3.44 mg L^−1^ vs. 5.97 mg L^−1^). However, the fucoxanthin content reported in our study is higher than in another study that cultivated *P. tricornutum* in an aerated 1 L reactor using different light intensities [33]. It is widely reported in the literature that light quality is the main element affecting the production of fucoxanthin [5,7,33]. However, this parameter was not taken into consideration in our study, as we only evaluated the effect of alternative media on the productivity of this carotenoid.

As reported by many authors, a microalgae-based refinery is more economical when it produces a wide range of products [3,22]. With a view to designing a biorefinery process starting from FBW, we also evaluated the carotenoid profile of the biomass obtained and the fatty acid profile. Fucoxanthin accounted for over 90% of total carotenoids detected, and a minor content of β-carotene. The obtained profile is very similar to those found in the literature, which show a predominance of fucoxanthin among the carotenoids extracted from *P. tricornutum* [34].

The biomass obtained proves to be a source of high-added-value elements such as carotenoids and PUFAs. In fact, we found that the EPA content of the optimized media after CCD was similar to the control (13% vs. 11% on TFA respectively). The EPA concentration observed in the biomass was in line with other studies found in the literature for *P. tricornutum* [26]. FBWs have been widely reported as optimal sources in the cultivation of omega-3-rich aquatic protists [2,35,36]. In order to compete with EPA from fish oil, many companies are focusing on the research of low-cost nutrient sources to use for their fermentation processes [2]. Therefore, nutrient recovery from food process wastewater is an important goal to achieve in order to reduce the production costs for the obtainment of omega-3 oil from microalgae, and at the same time reduce the organic load of these effluents [18]. In fact, the development of a food waste-based biorefinery through the growth of *P. tricornutum* would be able not only to generate added-value compounds but also to reduce the polluting load of the wastes used. In our case, no external nutrient was supplemented for the growth medium formulation but only FBW, therefore obtaining a growth model with high sustainability in economic terms and with low environmental impact.

## 4. Materials and Methods

### 4.1. Organism and Cultivation

*Phaeodactylum tricornutum* (SAG 1090-1a) was obtained from the Culture Collection of Algae at the University of Göttingen (SAG), Göttingen, Germany. Stock cultures of axenic microalgal strains were maintained routinely by regular sub-culturing at 4-week intervals on both liquid and agar slants of artificial seawater fortified with F/2 nutrients using the protocol of Guillard [37] at 20 °C ± 1. For the trials, 10% *v*/*v* of inoculum was placed in a 500 mL glass flask with 250 mL of working volume culture in aseptic conditions. Gas exchange and mixing in the cultures were provided by means of air bubbling (flow rate 0.8 *v*/*v* min^−1^) equipped with a filter of 0.22 µm to avoid culture contamination. All the trials were carried out in triplicate.

In the first screening, the growth performance of the BG11 medium was compared with standard F/2 Guillard. The BG-11 medium has the following composition: NaNO_3_ (1.5 g L^−1^), K_2_HPO_4_ (40 mg L^−1^), CaCl_2_·2H_2_O (30 mg L^−1^), Na_2_CO_3_ (19 mg L^−1^), MgSO_4_·7H_2_O (8 mg L^−1^), C_6_H_8_O_7_·H_2_O (7 mg L^−1^), ammonium ferric citrate (6 mg L^−1^), H_3_BO_3_ (3 mg L^−1^), MnCl_2_·4H_2_O (2 mg L^−1^), Na_2_EDTA·2H_2_O (0.7 mg L^−1^), Na_2_MoO_4_·2H_2_O (0.4 mg L^−1^), ZnSO_4_·7H_2_O (0.2 mg L^−1^), CuSO_4_·5H_2_O (0.1 mg L^−1^), and Co(NO_3_)_2_·6H_2_O (0.05 mg L^−1^). The Guillard F/2 medium has the following composition: NaNO_3_ (75 mg L^−1^), NaH2PO_4_·H_2_O (5 mg L^−1^), FeCl_3_·6H_2_O (3.15 mg L^−1^), Na_2_EDTA·2H_2_O (4.36 mg L^−1^), CuSO_4_·5H_2_O (9.8 mg L^−1^), Na_2_MoO_4_·2H_2_O (6.3 mg L^−1^), ZnSO_4_·7H_2_O (22 mg L^−1^), CoCl_2_·6H_2_O (10 mg L^−1^), MnCl_2_·4H_2_O (180 mg L^−1^), and vitamins thiamine hydrochloride (vitamin B1, 200 mg L^−1^), biotin (vitamin H, 0.1 mg L^−1^), and cyanocobalamin (vitamin B12, 1 mg L^−1^). The BG11 medium was prepared using seawater, the pH was adjusted to 8.0, and the mixtures were autoclaved at 121 °C for 15 min. Continuous illumination of 200 μmol photons m^−2^ s^−1^ was provided to the flasks [25].

### 4.2. Growth Parameters

The monitoring of the growth performances for control and treated samples were obtained through growth curves using standard gravimetric methods on daily aliquots of cultures. The dry weight (DW, g L^−1^) was obtained after centrifugation at 4695× *g* for 15 min and the pellet was rinsed twice to remove any residual salt. The pellet was dried at 75 °C until a constant weight was reached.

### 4.3. Food By-Products and Cheese Whey Pre-Treatments

CW samples were generously provided by a mozzarella cheese factory (Capurso Azienda Casearia srl, Italy) that concentrates CW using reverse osmosis to reduce the volume to be sent to disposal. The concentrated CW samples were taken from the accumulation tanks of the factory, aliquoted, and immediately frozen at −20 °C to prevent any fermentation. Prior to their utilization, the CW samples were filtered to remove large solid particulates.

CW samples were subjected to enzymatic hydrolysis following the protocols proposed in our previous study [19]. The protocol used was the following: 300 mL of CW was heated at 85 °C for 1 h in a thermostatic bath. After that, the samples were transferred on an orbital shaker set at 37 °C with the addition of 186 mg/L of food-grade lactase. Finally, the samples were heated at 90 °C for 5 min to inactivate the lactase.

After this first step of hydrolysis, the proteolysis phase began. The bottles were placed on an orbital shaker at 50 °C, 150 rpm, and 12.5 mL of protease from *Aspergillus oryzae* (Merck, Rome, Italy) were added, corresponding to about 16,000 LAPU Aminopeptidase units per liter of MSW. Proteolysis was carried out for 3 h. After this period, the enzyme was inactivated at 85 °C for 3 h. The samples obtained were frozen to prevent any fermentation.

CW was the only waste that was subjected to enzymatic hydrolysis, while BM and CSL were used without any pre-treatment.

### 4.4. Chemical Characterization of FBW

The ash content of the FBW was determined gravimetrically until reaching a constant weight in a muffle furnace at 550 °C. The protein content was evaluated by the Bradford assay [38] using bovine serum albumin (BSA) as the standard (Sigma, St. Louis, MO, USA) and a Shimadzu UV-1700 spectrophotometer (Kyoto, Japan) for the reading of the absorbance.

Lactose was determined spectrophotometrically following AOAC Method 2006.06 [39], while the determination of reducing sugars was obtained with the dinitrosalicylic assay (DNS) [40].

Free amino nitrogen content was estimated with the ninhydrin reaction method described by Lie et al. (1973) [41].

### 4.5. Alternative Growth Media Screening

The various FBWs under examination were tested by evaluating them alone or in combination with each other. BG11 was used as the standard reference medium. BM, CW, and CSL media have been formulated to have the same nitrogen concentration as the reference control (0.2 g L^−1^ of N). The formulations of FBW media tested were the following: CSL medium = 3 g L^−1^ of CSL; BM medium = 10 g L^−1^ of beet molasses; and CW medium = 30 mL L^−1^. All the FBW were supplemented with artificial seawater (ASW).

The pH was adjusted at 8.0 for all the media with NaOH 5 M. All the experiments were carried out in triplicate. The cultures were grown at 21 ± 1 °C, under continuous illumination of 200 μmol photons m^−2^ s^−1^ (light-emitting diode) and mixing provided by an air bubbling system (flow rate 0.8 *v*/*v* min^−1^) in a 500 mL airlift flask. The inoculum concentration for the experiment was 200 mg/L of biomass. The growth performances were estimated by daily measurements of biomass dry weight.

### 4.6. Response Surface Analysis and Formulation of Optimized Media

A new medium obtained from hydrolyzed CW, BM, and CSL was optimized using response surface methodology (RSM) in combination with central composite design (CCD). This method was applied to formulate the optimal combination of the three FBW to supplement with seawater in order to enhance the biomass and fucoxanthin production of *P. tricornutum*.

The RSM was performed by constructing a five-level full factorial central composite design (CCD). The design included star points, which represent extreme values (−α, α) for each input factor.

The mathematical relationship of the response (Y) to the significant independent variables X_1_, X_2_, and X_3_ is given by the following quadratic polynomial Equation (3):

(3) $$Y=\beta_{0}+\sum_{i=1}^{n}\beta_{i}X_{i}+\sum_{i=1}^{n}\beta_{ii}X_{i}^{2}+\sum_{i=1}^{n}\beta_{ij}X_{i}X_{j}$$

where Y is the predicted response; X*_i_* and X*_j_* are the coded values; β_0_ is the independent coefficient; β_*i*,*j*_ is the linear coefficient associated to each independent factor (X_*i*,*j*_) and β*_ij_* and β*_ii_* are the coefficients for interaction and quadratic effects respectively. ANOVA was used to establish the statistical validation of the polynomial equations generated.

### 4.7. Lipid Extraction and Fatty Acid Methyl Esters (FAMEs)

The extraction of the total amount of lipids was carried out according to a method reported by Cha et al. [42] with minor modifications. For that purpose, 0.1 g of a powdered microalga sample was extracted with 3.33 mL of concentrated HCl (37%). The mixture was agitated for 2 min and boiled twice at 100 °C for 20 min to cause cell disruption. The tubes were cooled to room temperature. Finally, the lipid fraction was extracted three times: once with 4 mL of hexane and twice with 2.5 mL of hexane. A transmethylation procedure using a previously established technology with some changes was used to create the fatty acid methyl esters (FAMEs) from the whole amount of previously obtained lipids [43]. An amount of 20 mg of lipid extract was mixed with 50 µL 2N KOH in methanol, 500 µL of n-hexane, and 500 µL of methylnonadecanoate (Sigma, St. Louis, MO, USA) as internal standard (1 mg/ mL). The mixture was vortexed for two minutes. Gas chromatography–mass spectrometry was used to analyze the upper layer supernatant (FAME extract) (GC–MS). Microalgal extracts were analyzed according to the method conditions established by Conde et al. [44]. The analyses were carried out by using an Agilent 7890A Gas chromatograph coupled to a Waters QUATTRO microTM mass spectrometer detector. A capillary column DB-5MS (30 m × 0.25 mm; f.t. 0.25 µm) purchased from Agilent Technologies (J&W Scientific, Folsom, CA, USA) was used for the separation of fatty acids. The oven temperature was 58 °C for 2 min, 25 °C min^−1^ to 160 °C, 2 °C min^−1^ to 210 °C, and 30 °C min^−1^ to 225 °C (held for 20 min). The operating parameters of the MS detector include an ionization energy of 70 eV and a scanning range of 50–550 *m*/*z*. The conditions were helium as carrier gas at 1.4 mL min^−1^, inlet temperature of 220 °C, detector temperature of 230 °C, and 2 µL of injection volume (splitless). Data were analyzed using MassLynx version 4.1 (Waters, San Jose, CA, USA).

### 4.8. Determination of Carotenoids in Microalgal Extracts by HPLC/MS Analysis

The extraction was accomplished using an ultrasonic bath (Bandelin, Sonorex, RK52, Berlin, Germany) that runs at a frequency of 35 kHz in accordance with the procedure previously described by Castro-Puyana et al. [45] with some modifications. Briefly, 10 mg of a sample of microalgal extract was added to 1.5 mL of ethanol containing 0.1% (*w*/*v*) of butylated hydroxytoluene. The mixture was centrifuged at 10,000 rpm for 10 min, at 4 °C. Before the analyses, the extracts were collected, filtered via 0.2 m nylon syringe filters, and then kept at −18 °C. Microalgal extracts were analyzed by UPLC Acquity coupled to a XEVO-TQ-S Triple quadrupole mass spectrometer (Waters Corporation, Milford, MA, USA). A YMC-C30 reversed-phase column (250 × 4.6 mm. 3 µm) was used for the separation of carotenoids. The mobile phases consisted of methanol with 5% water and 0.1% formic acid as mobile phase A and methyl tert-butyl ether as mobile phase B. The gradient conditions were 60% A to 0% A in 30 min with a flow rate of 1 mL min^−1^. Analysis parameters were arranged using a positive ion mode. The parameters of multiple reactions monitoring MRM transitions for all the standards are listed in Supplementary Materials (Table S1). Additional mass spectrometric parameters were as follows: source temperature was 150 °C, the desolvation temperature was 500 °C, the cone gas flow was 150 °C, the source offset was 30 V, the desolvation gas flow was 1000 L/h, the collision gas flow was 0.15 mL min^−1^, and the collision gas was argon. The data were acquired using MassLynx version 4.1 (Waters, San Jose, CA, USA). Violaxanthin, astaxanthin, canthaxanthin, and β-carotene were used as analytical standards for the quantification of carotenoids. The calibration curves were from the limit of quantification (LOQ) to 500–625 mg L^−1^. All calibration curves showed good linearity among different concentrations, and the determination coefficients were greater than 0.9918 in all cases. The limit of detection (LOD) was in the range of 0.02–2.06 µg L^−1^ and the LOQ was within 0.08–6.85 µg L^−1^ (Supplementary Materials Table S2).

### 4.9. Statistical Analysis

All the analyses were carried out in triplicate, and average values with standard deviation were reported. One-way ANOVA was applied using raw data to test for significant differences among the samples (significance level was always set at *p* < 0.05). Tukey’s test was used for post hoc analysis, when there were significant differences among the samples. The data were analyzed using IBM^©^ SPSS^©^ Statistics software Ver. 23 (SPSS, Inc., Chicago, IL, USA). RSM analysis was carried out using the Statistica 7.0 package (StatSoft, Tulsa, OK, USA).

## 5. Conclusions

Hydrolyzed CW, BM, and CSL were found to be excellent alternative nutrient sources for the production of biomass and fucoxanthin by *P. tricornutum*. The CW has been subjected to enzymatic hydrolysis in order to increase the bioavailability of the nutrients present. The amount of fucoxanthin produced was found to be significantly higher in molasses-based media and CSL with respect to the standard medium and CW-based medium.

Through CCD, a new medium for the cultivation of *P. tricornutum* was formulated by mixing the three FBWs to obtain the maximum amount of biomass and fucoxanthin yield. The best formulation was 3.3% *v*/*v* of CW, 2.3 g L^−1^ of molasses, and 2.24 g L^−1^ of corn steep liquor. A lipid fraction with 11% EPA on TFAs was also extracted from the obtained biomass, increasing the added value of the whole process.

The proposed biotechnological route for the valorization of dairy wastewater and food by-product could reduce the production cost of molecules of high economical interest, while at the same time mitigating the pollution and organic load derived from food wastewater disposal.

## Figures and Tables

**Table 1:** Chemical and physical characterization of food waste used.

Parameters	CW	hCW	CSL	BM
pH	5.3 ± 0.1	5.7 ± 0.1	3.6 ± 0.1	4.2 ± 0.2
Ash (g kg^−1^)	16.5 ± 0.4	14.2 ± 0.2	137.5 ± 2.2	81.9 ± 0.4
Dry weight (%)	21.9 ± 1.1	20.1 ± 0.4	62.59 ± 1.8	73.2 ± 0.8
N total (g kg^−1^)	6.7 ± 0.2	6.4 ± 0.2	48.45 ± 2.9	13.92 ± 0.08
Protein content (g kg^−1^)	28.8 ± 0.5	5.18 ± 0.3	305.83 ± 1.3	78.13 ± 1.1
Lactic acid (g kg^−1^)	1.5 ± 0.2	1.9 ± 0.1	121.2± 2.7	33.4 ± 1.2
Free amino nitrogen (g kg^−1^)	0.31 ± 0.06	3.19 ± 0.06	18.19 ± 0.6	0.11 ± 0.03
Total sugars (g kg^−1^)	201.2 ± 2.2	197.2 ± 3.1	35.1 ± 0.8	667.2 ± 4.5
Lactose (g kg^−1^)	196.8 ± 2.7	8.1 ± 1.1	n.d.	n.d.
Total P (mg kg^−1^)	145 ± 3.5	132 ± 1.3	18.2 ± 0.4	26.6 ± 1.1

CW = concentrated cheese whey; hCW = hydrolyzed cheese whey; CSL = corn steep liquor; BM = beet molasses; n.d. = not detected. Values expressed as mean (*n* = 3) ± SD.

**Table 2:** RSM–CCD design and results of biomass growth and fucoxanthin yield optimization with a combination of CW, BM, and CSL.

Run	Factor Assignment			Responses (Y)	
	X_1_ (BM)	X_2_ (CW)	X_3_ (CSL)	Biomass DW (g L^−1^)	Fucoxanthin Yield (mg L^−1^)
1	1.9	25	1.4	2.41	2.92
2	1.9	25	2.379	2.54	3.22
3	1.9	25	0.421	1.90	1.87
4	1.9	0.505	1.4	1.91	2.33
5	3.696	25	1.4	2.18	3.67
6	1.9	25	1.4	2.25	2.50
7	1.9	49.495	1.4	2.31	2.84
8	0.103	25	1.4	1.89	2.23
9	3	40	2	1.91	2.53
10	0.8	40	0.8	2.14	2.40
11	3	40	0.8	2.21	3.07
12	1.9	25	1.4	2.21	2.67
13	0.8	10	2	1.75	3.09
14	0.8	40	2	2.16	2.45
15	3	10	2	1.94	3.22
16	0.8	10	0.8	1.51	1.96
17	1.9	25	1.4	2.07	2.42
18	1.9	25	1.4	2.31	2.74
19	3	10	0.8	1.66	2.39
20	1.9	25	1.4	2.27	2.67

Coded values: X_1_ = beet molasses (g/L); X_2_ = hydrolyzed cheese whey (mL/L); X_3_ = corn steep liquor (g/L); the five levels (−α, −1, 0, +1, and +α) set for BM were 0.103, 0.8, 1.9, 3, and 3.696 g L^−1^, while for CW they were 0.505, 10, 25, 40, and 49.495 mL L^−1^, respectively.

**Table 3:** Analysis of variance for biomass production using coded values and regression equation.

Source	DF ^a^	Adj SS ^b^	Adj MS ^c^	F-Value	*p*-Value
Model	10	1.118	0.1118	5.45	0.009
Blocks	1	0.127	0.1272	6.21	0.034
Linear	3	0.519	0.1731	8.44	0.006
Molasses	1	0.029	0.0296	1.44	0.26
CW	1	0.366	0.3665	17.87	0.002
CSL	1	0.123	0.1232	6.01	0.037
Square	3	0.347	0.1159	5.65	0.019
Molasses ×Molasses	1	0.217	0.2171	10.59	0.01
CW × CW	1	0.130	0.1301	6.34	0.033
CSL × CSL	1	0.042	0.0424	2.07	0.184
2-Way Interaction	3	0.123	0.0412	2.01	0.183
BM × CW	1	0.034	0.0340	1.66	0.23
BM × CSL	1	0.0099	0.0099	0.48	0.504
CW × CSL	1	0.0796	0.0796	3.88	0.08
Error	9	0.1845	0.020		
Lack-of-Fit	5	0.13928	0.027856	2.46	0.202
Pure Error	4	0.04531	0.011328		
Total	19	1.30267			

R^2^ = 97.12 (^a^ DF, degree of freedom; ^b^ SS, sum of squares; ^c^ MS, mean squares; F, probability of distribution; *p*, probability).

**Table 4:** Analysis of variance for fucoxanthin production using coded values and regression equation.

Source	DF ^a^	Adj SS ^b^	Adj MS ^c^	F-Value	*p*-Value
Model	10	2.829	0.283	3.900	0.021
Blocks	1	0.018	0.018	0.240	0.633
Linear	3	1.851	0.617	8.490	0.005
Molasses	1	0.858	0.858	11.810	0.007
CW	1	0.041	0.041	0.560	0.473
CSL	1	0.953	0.953	13.120	0.006
Square	3	0.151	0.050	0.690	0.579
Molasses × Molasses	1	0.084	0.084	1.160	0.309
CW × CW	1	0.019	0.019	0.260	0.626
CSL × CSL	1	0.037	0.037	0.510	0.492
2-Way Interaction	3	0.810	0.270	3.710	0.055
BM × CW	1	0.011	0.011	0.150	0.704
BM × CSL	1	0.127	0.127	1.750	0.218
CW × CSL	1	0.671	0.671	9.240	0.014
Error	9	0.654	0.073		
Lack-of-Fit	5	0.507	0.101	2.770	0.212
Pure Error	4	0.146	0.037		
Total	19	3.483			

R^2^ = 96.22 (^a^ DF, degree of freedom; ^b^ SS, sum of squares; ^c^ MS, mean squares; F, probability of distribution; *p*, probability).

**Table 5:** Table of quantification of carotenoids in *P. tricornutum* biomass by HPLC-MS.

Medium	Fucoxanthin	Violaxanthin	Antheraxanthin	Lutein	β-carotene
BG11	665.1 ± 7.8 ^b^	n.d.	0.64 ± 0.1	0.16 ± 0.01	7.42 ± 0.3 ^a^
Guillard F/2	394 ± 1.5 ^a^	n.d.	0.95 ± 0.05	0.13 ± 0.02	3.89 ± 0.5 ^a^
BM medium	1221.1 ± 8.2 ^c^	n.d.	0.66 ± 0.03	n.d.	15.85 ± 0.8 ^b^
CW medium	410.3 ± 2.4 ^a^	n.d.	0.48 ± 0.02	0.35 ± 0.03	36.8 ± 1.1 ^c^
CSL medium	808.2 ± 4.1 ^d^	0.70 ± 0.05	0.34 ± 0.01	n.d.	34.23 ± 1.2 ^c^
Optimized medium	1785.7 ± 5.1 ^e^	0.41 ± 0.01	0.68 ± 0.03	0.25 ± 0.03	47.6 ± 0.2 ^d^

Data are given as µg/g of dried biomass. CW = concentrated cheese whey; CSL = corn steep liquor; BM = beet molasses; n.d. = not detected. Values are expressed as means ± SD (*n* = 3). Different letters in the same column mean a significant difference (*p* < 0.05).

## Data Availability

The data presented in this study are available on request from the corresponding author.

## Supplementary Materials

The following supporting information can be downloaded at: https://www.mdpi.com/article/10.3390/md21030190/s1. Table S1. Parameters for The MRM transitions. Table S2. Calibration curves of standards for the determination of carotenoid in microalgae.
